# 

**Published:** 1884-09

**Authors:** 


					﻿THIRTY-FIRST YEAR.
HALL’S
JOURNAL
OF
HEALTH
Guaranteed Circulation 200,000 Copies in 12 Months.
E. H. GIBBS, AM, M.D., Editor,
No. 21 CLINTON PLACE EIGHTH STREET NEW YORK.
TERMS: $1 a Year.
Single number, 10 cts.
				

